# Clinical Prognostic Modeling and Paired Blood–CSF Metabolomic Profiling for Outcome Prediction in Isolated Moderate-to-Severe Traumatic Brain Injury: Implications for Neurocritical Care Management

**DOI:** 10.3390/jcm15124592

**Published:** 2026-06-13

**Authors:** Zhuoying Du, Qifang Chen, Yuzhuo Wang, Pengfei Fu, Jin Hu, Gang Wu, Weijian Yang

**Affiliations:** 1 Department of Neurosurgery, Huashan Hospital, Shanghai Medical College, Fudan University, Shanghai 200040, China; 2 National Center for Neurological Disorders, Shanghai 200040, China; 3 Shanghai Key Laboratory of Brain Function Restoration and Neural Regeneration, Shanghai 200040, China; 4 Neurosurgical Institute of Fudan University, Shanghai 200040, China; 5 Shanghai Clinical Medical Center of Neurosurgery, Shanghai 200040, China; 6 Department of Nursing, Huashan Hospital, Shanghai Medical College, Fudan University, Shanghai 200040, China

**Keywords:** traumatic brain injury, neurocritical care, metabolomics, prognosis, outcome prediction, lactate, Glasgow coma scale, biomarker

## Abstract

**Objectives:** This study aimed to develop a prognostic model for isolated moderate-to-severe traumatic brain injury (TBI) (Glasgow Coma Scale [GCS] ≤ 12) using readily available variables and to explore paired blood–cerebrospinal fluid (CSF) metabolomic signatures. **Methods:** Consecutive TBI patients admitted between January 2019 and June 2025 were retrospectively analyzed. Multivariate logistic regression with bootstrap internal validation identified predictors of 6-month unfavorable outcome and in-hospital mortality. Untargeted metabolomics was performed on paired blood and CSF samples from 30 matched male patients. **Results:** Among 405 patients, 266 (65.7%) had unfavorable outcomes and 54 (13.3%) died in hospital. Rotterdam CT Score (odds ratio [OR] 10.59, 95% confidence interval [CI] 6.19–18.14), initial lactate (OR 1.81, 95% CI 1.38–2.36), and blood glucose (OR 1.40, 95% CI 1.21–1.64) predicted unfavorable outcome (area under the receiver operating characteristic curve [AUC] 0.97). GCS motor score (OR 0.50, 95% CI 0.37–0.66), initial lactate (OR 1.57, 95% CI 1.31–1.91), and follow-up lactate (OR 1.57, 95% CI 1.34–1.88) predicted mortality (AUC 0.96). Blood metabolomics revealed enrichment in energy and lipid metabolism pathways. CSF metabolomics highlighted neurotransmitter pathway dysregulation and neuroinflammatory markers, with depleted kynurenic acid in both biofluids. **Conclusions:** Readily available admission variables enable early bedside risk stratification in TBI. Metabolomic profiling links unfavorable outcomes to systemic energy–lipid dysregulation and central neuroinflammatory–neurotransmitter disturbances, with the tryptophan–kynurenine axis as a potential therapeutic target for neuroprotective strategies.

## 1. Introduction

Traumatic brain injury (TBI) remains a major public health challenge and a leading cause of death and long-term disability worldwide [1]. Patients with isolated moderate-to-severe TBI (GCS ≤ 12) frequently develop devastating outcomes despite advances in neurosurgery, neurocritical care, and multimodal monitoring [2]. In this study, we focus on this specific patient population and refer to them as TBI patients throughout the remainder of the manuscript. This heterogeneity reflects the complex interplay between the initial mechanical insult and secondary injury cascades, including cerebral hypoxia, mitochondrial dysfunction, oxidative stress, excitotoxicity, inflammation, and metabolic failure [3,4].

Early prognostic assessment is particularly important in TBI, in which the confounding effects of extracranial trauma are minimized. Early identification of risk factors for poor prognosis is essential for individualized treatment planning and resource allocation. Traditional prognostic variables such as age, admission Glasgow Coma Scale (GCS), pupillary findings, and imaging features remain clinically valuable, but they do not fully capture the metabolic and biological complexity of evolving secondary injury [5]. Increasing attention has therefore been directed toward circulating and central nervous system biomarkers that may more directly reflect the biological response to injury [6,7].

Metabolomics offers a complementary strategy for examining the biochemical landscape of TBI [8]. Untargeted metabolomics can capture broad metabolic disturbances across amino acid metabolism, lipid remodeling, oxidative stress pathways, neurotransmitter metabolism, and mitochondrial function. Blood metabolomics may reflect systemic injury response, whereas cerebrospinal fluid (CSF) metabolomics may more directly reveal central pathophysiological changes [9]. Yet studies integrating routine clinical predictors such as lactate with paired blood and CSF metabolomics in TBI remain limited.

Several critical gaps remain in the current literature. First, few studies have simultaneously profiled both blood and CSF metabolomes in the same patient cohort, limiting understanding of compartment-specific metabolic responses and blood–brain barrier dynamics. Second, most existing studies have focused on severe TBI (GCS ≤ 8), with limited data on moderate TBI (GCS 9–12), which represents a substantial proportion of patients requiring intensive care [10]. From a clinical management perspective, early and accurate prognostic stratification is fundamental to optimizing neurocritical care strategies in TBI. Identification of high-risk patients at admission may guide the intensity of monitoring, inform decisions regarding invasive intracranial pressure surveillance, and facilitate timely allocation of critical care resources.

To address these gaps, we conducted a retrospective cohort study of 405 TBI patients, integrating clinical phenotyping with exploratory metabolomic analysis of paired blood and CSF samples to: (1) develop and internally validate a clinical prognostic model for 6-month unfavorable outcome and in-hospital mortality in patients with TBI using readily available admission physiologic, biochemical variables, with imaging-based indices; (2) perform exploratory untargeted metabolomic profiling of paired blood and CSF samples to identify pathway-level alterations associated with outcome; and (3) integrate clinical and metabolomic findings to generate mechanistic hypotheses for future investigation.

## 2. Materials and Methods

### 2.1. Study Design and Participants

This retrospective cohort study was conducted at Huashan Hospital, Fudan University, a national center for neurological disorders. This study was conducted in accordance with the ethical standards of the Declaration of Helsinki. For the retrospective clinical component, patient data were anonymized, and the requirement for informed consent was waived by the ethics committee. Informed consent was obtained from the patients’ legal representatives for the prospective collection of blood and CSF samples for metabolomics analysis. The study protocol was approved by the Ethics Committee of Huashan Hospital, Fudan University (approval number: 2022-062, 17 February 2022).

This retrospective clinical cohort study included patients aged ≥18 years who were admitted for the first time due to TBI (GCS score ≤ 12) between January 2019 and June 2025, and who presented to the emergency department within 24 h of injury. The diagnosis of TBI was confirmed on admission by computed tomography (CT) scan in the emergency department, and no other body region had an Abbreviated Injury Scale score ≥ 3. Exclusion criteria were: (1) pregnancy; (2) history of central nervous system disease, immunodeficiency, diabetes, or coagulation disorders; (3) history of significant hepatic or renal disease; (4) immunosuppressive or anti-inflammatory treatment upon admission; and (5) loss to follow-up or incomplete data. To minimize potential confounding from extracranial hemorrhage, patients with clinically significant scalp lacerations requiring operative hemostasis or those with documented hemodynamic instability attributable to external blood loss were also excluded.

### 2.2. Data Collection

Demographic data (age and sex), injury characteristics, GCS score, Glasgow Outcome Scale (GOS) score, surgical details, antithrombotic therapy status on admission, and laboratory data were collected from electronic medical records. Rotterdam CT Score data were independently extracted by three researchers using the picture archiving and communication system (PACS). Laboratory data included: coagulation parameters (international normalized ratio [INR], prothrombin time [PT], activated partial thromboplastin time [APTT], fibrinogen [FIB], thrombin time [TT], d-dimer [DDI], fibrin degradation products [FDPs]), complete blood count (white blood cell [WBC] count and differential WBC count, red blood cell [RBC], hemoglobin [HB], hematocrit [HCT], platelet [PLT]), serum electrolytes (sodium, potassium, calcium, phosphorus), total protein, albumin, lactate, blood glucose, c-reactive protein, procalcitonin.

Emergency department laboratory data on admission were defined as “Initial [values]” (e.g., “Initial White blood cell”), whereas laboratory data reported as “[value]” (e.g., “White blood cell”) represented reassessments on the first postoperative day. The delta value is defined as the emergency laboratory value minus the value on the first postoperative day. The delta value ratio (%) is then calculated as (emergency laboratory value − value on the first postoperative day)/emergency laboratory value × 100%.

### 2.3. Outcome Measures

Global neurological function at 6 months after discharge was assessed using the Glasgow Outcome Scale (GOS), which was dichotomized into favorable (GOS 4–5) and unfavorable (GOS 1–3) outcomes for the primary outcome [11]. The secondary outcome was in-hospital mortality. GOS was assessed by trained research nurses through structured telephone interviews or outpatient clinic visits at 6 months post-injury. Patients transferred to a local hospital with unstable vital signs or those whose families opted to discontinue treatment were classified as in-hospital deaths. The dataset underwent standard preprocessing to ensure data integrity and confidentiality.

### 2.4. Metabolomic Profiling

For the metabolomic analysis, 30 matched pairs of male patients were identified according to age, injury mechanism, time from injury to sampling, and admission GCS score. Female hormones, including estrogen and progesterone, modulate steroidogenesis, electrolyte balance, and neurotrophic factor expression—mechanisms that may confer resilience to brain injury and facilitate faster neurological recovery in females. To eliminate potential confounding from sex-related hormonal influences, only male patients were included, resulting in a cohort of 30 matched male pairs. Each pair comprised one patient with a favorable outcome and one with an unfavorable outcome based on the GOS at 6 months after discharge.

Blood samples obtained at emergency department admission were subjected to metabolomic profiling. CSF samples were collected during intraventricular catheter insertion for external ventricular drainage or intracranial pressure monitoring, as part of standard clinical care within 24 h of injury. In 38 (63.3%) patients, the catheter was placed as the initial procedure, whereas in 22 (36.7%) patients, it was inserted following surgical hematoma evacuation. This heterogeneity in CSF sampling timing relative to surgical intervention may introduce variability in metabolomic profiles through blood–brain barrier disruption, inflammatory activation, and mechanical manipulation, and is acknowledged as a limitation. Both blood and CSF samples were obtained from the same individuals. Samples were processed and stored at −80 °C within 30 min of collection.

Untargeted metabolomic profiling was performed using a high-resolution liquid chromatography–mass spectrometry (LC-MS) system (Thermo Scientific™, Waltham, MA, USA). Data processing, including metabolite identification and quantification, was carried out using Compound Discoverer™ 3.3 software with integrated MS/MS spectral libraries. The analysis was conducted by Shanghai Personalbio Technology Co., Ltd. (Shanghai, China).

### 2.5. Statistical Analysis

Continuous variables were expressed as mean ± standard deviation (SD) or median (interquartile range [IQR]), as appropriate, and compared using Student’s *t*-test or the Mann–Whitney U test. Categorical variables were presented as frequencies (percentages) and compared using the chi-square test. Least absolute shrinkage and selection operator (LASSO) regression was applied to identify prognostic factors among the 405 TBI patients. The optimal regularization parameter λ was selected via ten-fold cross-validation. Multivariable logistic regression analysis was subsequently performed to confirm the variables retained by LASSO.

The predictive performance of individual variables and combined models for mortality and unfavorable outcomes was evaluated using receiver operating characteristic (ROC) curve analysis, with emphasis on clinical utility and potential implementation in neurosurgical practice. Optimal cut-off values were determined using the Youden index. Sensitivity, specificity, positive predictive value (PPV), and negative predictive value (NPV) were reported. Odds ratios (ORs) with 95% confidence intervals (CIs) were calculated. Multicollinearity was assessed using the variance inflation factor (VIF), with a VIF < 5 considered acceptable. Non-linearity of continuous predictors was assessed using restricted cubic splines (RCSs) with knots at the 25th, 50th, and 75th percentiles. LASSO regression with 10-fold cross-validation was employed as the first stage for variable selection from 62 candidate predictors, leveraging L1 regularization to handle high-dimensional data and address multicollinearity by shrinking redundant coefficients to zero. The lambda.1se criterion was applied to select a parsimonious set of predictors. Variables retained by LASSO were subsequently entered into multivariable logistic regression with bidirectional stepwise selection (based on AIC) as the second stage to confirm independent associations and obtain final ORs with 95% CIs. This two-stage approach combines regularization-based variable selection with traditional inference-based confirmation. To evaluate the incremental predictive value of lactate beyond established prognostic frameworks, we constructed an IMPACT-Lab proxy model using all available IMPACT variables (Age, GCS, Rotterdam CT Score as proxy for CT Classification, Glucose, and Hemoglobin). Incremental value was assessed using the Net Reclassification Index (NRI), Integrated Discrimination Improvement (IDI), and likelihood ratio test. All clinical analyses were performed using R version 4.3.3.

Internal validation was performed using bootstrap resampling (1000 iterations) following Harrell’s method [12]. In each iteration, a bootstrap sample of equal size was drawn with replacement from the original dataset. The prediction model was refitted on the bootstrap sample and evaluated on both the bootstrap sample (apparent performance) and the original sample (test performance). The difference in performance between the bootstrap and original samples defined the optimism. The optimism-corrected performance was calculated as the apparent performance minus the mean optimism across all iterations. Calibration slope was estimated by regressing the outcome on the logit of predicted probabilities in the original sample using the bootstrap-derived model. Brier score was calculated to assess the overall accuracy of predicted probabilities.

For the metabolomic data, dimensionality reduction analyses, including principal component analysis (PCA), partial least squares discriminant analysis (PLS-DA), and orthogonal PLS-DA (OPLS-DA), were conducted using the R package ropls. Model overfitting was assessed by permutation testing. R2X and R2Y represent the proportions of variance explained in the X and Y matrices, respectively, while Q2 reflects the predictive ability of the models. Variable importance in projection (VIP) scores from OPLS-DA, *p*-values, and fold change (FC) were calculated to quantify the discriminatory power of individual metabolite features on sample classification. Metabolites with *p* < 0.05 and VIP > 1 were considered statistically significant.

Hierarchical clustering of differential metabolite abundances was performed using the pheatmap package (version 1.0.12) in R. Box plots illustrating abundance patterns were created using ggplot2 (version 3.4.1). ROC curves were plotted using the pROC package (version 1.18.2). Functional analysis of differential metabolites was conducted using Kyoto Encyclopedia of Genes and Genomes (KEGG) enrichment analysis via the clusterProfiler package (version 4.6.0). Differential abundance scores were calculated to capture the global trend of all differential metabolites within each pathway.

## 3. Results

### 3.1. Patient Characteristics

A total of 405 TBI patients were included (299 [73.8%] male; median age 57 years). The most common mechanism was road traffic accident (248, 61.2%). On admission, 226 (55.8%) had GCS ≤ 8 and 179 (44.2%) had GCS 9–12. At 6 months, 139 (34.3%) achieved favorable outcomes, 266 (65.7%) had unfavorable outcomes, and 54 (13.3%) died in hospital (Table 1).

### 3.2. Comparative Analysis: Favorable Versus Unfavorable Outcome

Comparative analysis between favorable (*n* = 139) and unfavorable outcomes (*n* = 266) is presented in Table 2 (Key variables) and Supplementary Table S1 (All variables). Patients with unfavorable outcomes were significantly older (58.5 ± 15.8 vs. 53.5 ± 15.1 years, *p* = 0.002). Sex, TBI etiology, surgical approach, and comorbidities did not differ between groups.

**Neurological Severity.** Admission GCS scores were significantly lower in the unfavorable outcome group (median 7 [5, 9] vs. 10 [9, 11], *p* < 0.001), with all three components comparably reduced.

**Neuroimaging.** Rotterdam CT scores were significantly higher in the unfavorable outcome group (median 6 [5, 6] vs. 3 [3, 4], *p* < 0.001), driven by differences in basal cistern status, midline shift, and epidural mass lesion.

**Coagulation parameters.** The unfavorable outcome group exhibited coagulopathy, with significantly higher INR (1.1 ± 0.3 vs. 1.0 ± 0.2, *p* = 0.03), lower FIB (2.5 ± 1.3 vs. 2.8 ± 1.1, *p* = 0.02), prolonged TT (17.9 ± 4.0 vs. 17.2 ± 2.5 s, *p* = 0.03), and markedly elevated D-dimer (37.0 ± 26.5 vs. 23.7 ± 23.6 mg/L, *p* < 0.001) and FDP (128.0 ± 163.3 vs. 64.58 ± 89.6 mg/L, *p* < 0.001).

**Hematologic parameters.** On admission, the unfavorable outcome group had lower RBC (4.2 ± 0.6 vs. 4.4 ± 0.6 × 10^12^/L, *p* = 0.004), hemoglobin (129.6 ± 17.9 vs. 135.1 ± 16.4 g/L, *p* = 0.003), and HCT (38.0 ± 5.3% vs. 39.7 ± 4.7%, *p* = 0.002), alongside higher WBC and neutrophil counts (*p* < 0.001 and *p* = 0.003, respectively). These differences persisted during hospitalization, with greater delta changes in RBC, hemoglobin, and HCT (all *p* < 0.001), reflecting more pronounced hemodilution or blood loss.

**Metabolic Parameters.** Lactate was significantly higher in the unfavorable outcome group both on admission (4.0 ± 2.2 vs. 1.7 ± 1.6 mmol/L, *p* < 0.001) and during follow-up (3.3 ± 3.4 vs. 1.6 ± 1.1 mmol/L, *p* < 0.001). Admission and follow-up glucose levels were similarly elevated (9.5 ± 2.8 vs. 8.6 ± 2.8, *p* = 0.003; 10.0 ± 3.1 vs. 7.99 ± 1.7 mmol/L, *p* < 0.001). Follow-up total protein and albumin were significantly lower (both *p* < 0.001), with greater delta changes in total protein (*p* = 0.01) and its delta rate (*p* = 0.006). Procalcitonin was markedly elevated in the unfavorable outcome group (1.8 ± 2.0 vs. 0.6 ± 0.7 ng/mL, *p* < 0.001).

### 3.3. Factors Associated with Unfavorable Outcome

**Figure 1:**
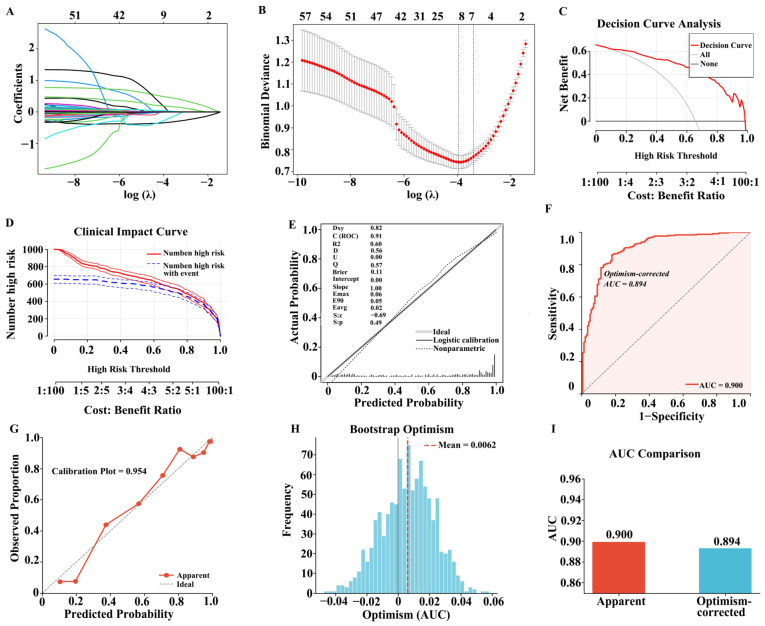
Prognostic model for unfavorable outcome. (**A**) LASSO coefficient profiles. (**B**) Cross-validation for selection of the optimal penalty parameter (λ). (**C**) Decision curve analysis demonstrating favorable clinical net benefit. (**D**) Clinical impact curve showing the number of patients classified as high-risk and the corresponding true positive cases across threshold probabilities. (**E**) Calibration plot showing good agreement between predicted and observed probabilities. (**F**) Internal bootstrap validation of the prognostic model for unfavorable outcome: ROC curve demonstrating an apparent AUC of 0.900. (**G**) Calibration plot from internal bootstrap validation comparing predicted probabilities with observed proportions of unfavorable outcomes; the dashed diagonal line represents perfect calibration (slope = 0.954). (**H**) Distribution of optimism (difference in AUC between bootstrap-resampled and original datasets) across 1000 bootstrap iterations; the red dashed line indicates mean optimism (0.0062). (**I**) Comparison of apparent and optimism-corrected AUC (0.894) following internal bootstrap validation. Abbreviations: LASSO, least absolute shrinkage and selection operator; ROC, receiver operating characteristic; AUC, area under the curve; DCA, decision curve analysis.

LASSO regression was applied to identify factors associated with unfavorable outcomes among the 405 TBI patients. The model was fitted under a binomial distribution family with 62 candidate clinical and laboratory predictors. Under the more parsimonious lambda.1se criterion (i.e., the largest λ within one standard error of the minimum), the model retained only three variables: Rotterdam CT score, initial lactate, and blood glucose (Figure 1A,B).

In a multivariable logistic regression analysis, the above three independent factors were retained in the final model after bidirectional stepwise selection (Table 3). Higher Rotterdam CT Score was significantly associated with unfavorable outcomes (OR 10.59, 95% CI: 6.19–18.14, *p* < 0.001). Higher initial lactate was significantly associated with unfavorable outcomes (OR 1.81, 95% CI: 1.38–2.36, *p* < 0.001). Elevated blood glucose also emerged as an independent risk factor for unfavorable outcome (OR 1.40, 95% CI: 1.21–1.64, *p* < 0.001). All analyses were based on complete cases, with no missing data included in the model.

In ROC analysis, Rotterdam CT score demonstrated the highest discriminative power (AUC 0.93) compared to initial Lactate (AUC 0.86) and Blood glucose (AUC 0.73) alone. The combination of all the three variables yielded an AUC of 0.97, representing an excellent predictive model for clinical use (Table 4). Decision curve analysis (DCA) and the clinical impact curve demonstrated that the model exhibited favorable clinical utility. The calibration curve indicated strong concordance between the predicted probabilities and the actual observed outcomes (Figure 1C–E).

Bootstrap internal validation (1000 iterations) demonstrated minimal overfitting for the unfavorable outcome model. The apparent AUC of 0.900 decreased to an optimism-corrected AUC of 0.894 (mean optimism 0.0062, 95% CI −0.0271 to 0.0365). The calibration slope was 0.954 (ideal value 1.0), and the Brier score was 0.114 (Figure 1F–I). These results indicate negligible overfitting and support the robustness of the prognostic model.

To evaluate whether lactate provides incremental prognostic value beyond established frameworks, we constructed an IMPACT-Lab proxy model using available variables (Supplementary Table S2). For unfavorable outcome prediction, adding initial lactate to the IMPACT-Lab proxy increased the AUC from 0.957 to 0.967 (NRI = 0.832, IDI = 0.059, both *p* < 0.001; likelihood ratio test χ^2^ = 27.76, *p* = 1.37 × 10^−7^). Notably, when lactate was included, glucose lost its independent significance (*p* = 0.233), while lactate remained highly significant (OR = 1.83, *p* < 0.001), suggesting that lactate captures metabolic derangement more effectively than glucose in a comprehensive model.

### 3.4. Clinical Characteristics Between Survivors and Non-Survivors

Comparative analysis between survivors (*n* = 351) and non-survivors (*n* = 54) is presented in Table 5 (Key variables) and Supplementary Table S3 (All variables). Non-survivors tended to be older (60.4 ± 18.3 vs. 56.2 ± 15.2 years, *p* = 0.07), with no significant differences in sex, TBI etiology, antithrombotic use, or comorbidities.

**Neurological Severity.** Non-survivors had lower admission GCS (median 4 [3, 6] vs. 8 [7, 11], *p* < 0.001).

**Neuroimaging.** Rotterdam CT scores were higher in the non-survivors (median 6 [6, 6] vs. 5 [4, 6], *p* < 0.001), driven by differences in basal cistern status, midline shift, and Intraventricular blood or traumatic subarachnoid hemorrhage (tSAH).

**Coagulation parameters.** Coagulation profiles revealed significant coagulopathy in non-survivors: elevated INR (1.2 ± 0.5 vs. 1.1 ± 0.1, *p* = 0.01), APTT (29.2 ± 16.3 vs. 24.6 ± 4.1 s, *p* = 0.04), and TT (20.6 ± 7.3 vs. 17.2 ± 2.3 s, *p* = 0.001), with markedly decreased fibrinogen (1.8 ± 1.1 vs. 2.7 ± 1.2 g/L, *p* < 0.001) and elevated D-dimer and FDP (both *p* < 0.001), consistent with consumptive coagulopathy.

**Hematologic parameters.** Non-survivors developed more pronounced anemia and thrombocytopenia during hospitalization (both *p* < 0.001).

**Metabolic Parameters.** Non-survivors exhibited markedly elevated admission lactate (6.4 ± 2.5 vs. 2.7 ± 1.9 mmol/L, *p* < 0.001) that persisted, alongside higher glucose at admission and throughout hospitalization (both *p* ≤ 0.01). Total protein and albumin declined more steeply (delta *p* < 0.001), and procalcitonin was elevated (2.6 ± 2.0 vs. 1.2 ± 1.6 ng/mL, *p* < 0.001), suggesting systemic inflammatory response.

### 3.5. Factors Associated with In-Hospital Mortality

**Figure 2:**
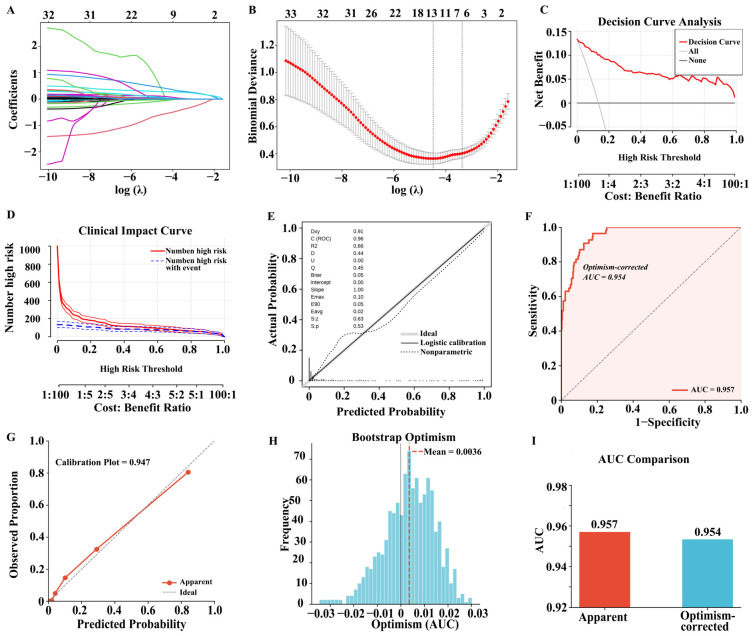
Prognostic model for in-hospital mortality. (**A**) LASSO coefficient profiles. (**B**) Cross-validation for selection of the optimal penalty parameter (λ). (**C**) Decision curve analysis demonstrating favorable clinical net benefit. (**D**) Clinical impact curve showing the number of patients classified as high-risk and the corresponding true positive cases across threshold probabilities. (**E**) Calibration plot showing good agreement between predicted and observed probabilities. (**F**) Internal bootstrap validation of the prognostic model for in-hospital mortality: ROC curve demonstrating an apparent AUC of 0.957. (**G**) Calibration plot from internal bootstrap validation comparing predicted probabilities with observed proportions of in-hospital mortality; the dashed diagonal line represents perfect calibration (slope = 0.947). (**H**) Distribution of optimism (difference in AUC between bootstrap-resampled and original datasets) across 1000 bootstrap iterations; the red dashed line indicates mean optimism (0.0036). (**I**) Comparison of apparent and optimism-corrected AUC (0.954) following internal bootstrap validation. Abbreviations: LASSO, least absolute shrinkage and selection operator; ROC, receiver operating characteristic; AUC, area under the curve; DCA, decision curve analysis; GCS M, motor component of the Glasgow Coma Scale.

LASSO regression was applied to identify factors associated with in-hospital mortality among 405 TBI patients. The model was fitted under a binomial distribution family with 62 candidate clinical and laboratory predictors. Under the more conservative λ-1se criterion—which balances predictive accuracy with model simplicity—only three variables retained non-zero coefficients: GCS-M, Initial Lactate, and follow-up lactate. All other covariates were shrunk to zero under both criteria, suggesting limited independent association with mortality risk after accounting for multicollinearity and overfitting through L1 regularization. These findings highlight GCS motor score and lactate levels (both initial and subsequent measurements) as the most robust and stable predictors of in-hospital mortality in this TBI cohort (Figure 2A,B).

In multivariable logistic regression analysis including all 405 patients (using stepwise selection with both forward and backward directions), after adjusting for other covariates, both follow-up lactate and initial lactate were significantly associated with increased risk of in-hospital mortality (OR = 1.57, 95% CI: 1.34–1.88, *p* < 0.001; OR = 1.57, 95% CI: 1.31–1.91, *p* < 0.001, respectively). GCS-M was significantly inversely associated with mortality (OR = 0.50, 95% CI: 0.37–0.66, *p* < 0.001). All three variables remained in the final model, suggesting they are independent predictors of in-hospital death among TBI patients. The data are shown in Table 6.

For in-hospital mortality (Table 7), follow-up lactate demonstrated the highest discrimination (AUC 0.85, 95%CI 0.78–0.91, cut-off 4.15 mmol/L, sensitivity 69%, specificity 90%). GCS-M showed comparable performance (AUC 0.84, 95%CI 0.79–0.89, cut-off 3, sensitivity 78%, specificity 80%). Initial lactate also performed well (AUC 0.81, 95%CI 0.75–0.87, cut-off 3.44, sensitivity 91%, specificity 70%). The combined model incorporating all predictors achieved excellent discrimination (AUC 0.96), with decision curve analysis demonstrating clinical net benefit across a wide range of threshold probabilities (Figure 2C–E).

Bootstrap internal validation (1000 iterations) confirmed excellent generalizability of the mortality model. The apparent AUC of 0.957 decreased to an optimism-corrected AUC of 0.954 (mean optimism 0.0036, 95% CI −0.0185 to 0.0228). The calibration slope was 0.948, and the Brier score was 0.053 (Figure 2F–I). These results indicate minimal overfitting and support the robustness of the mortality prediction model.

For mortality prediction, we constructed an IMPACT-Lab proxy model using available variables (Supplementary Table S4). Adding both initial and follow-up lactate increased the AUC from 0.875 to 0.960 (NRI = 1.268, IDI = 0.270, both *p* < 0.001). Rotterdam CT Score did not provide incremental value beyond lactate (*p* = 0.863), consistent with our LASSO-based variable selection. These results demonstrate that lactate provides meaningful incremental prognostic value beyond a comprehensive IMPACT-based model, particularly for mortality prediction.

### 3.6. Exploratory Untargeted Metabolomics of Emergency Blood Samples

**Figure 3:**
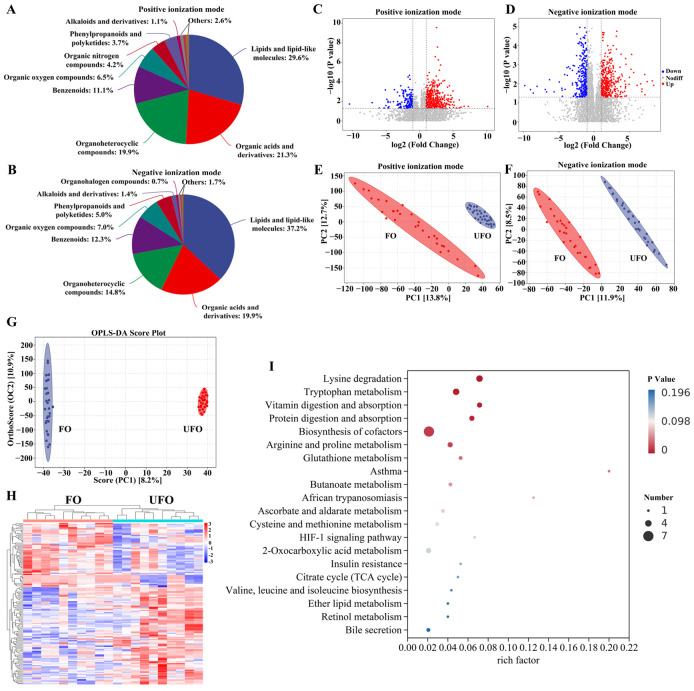
Non-targeted metabolomics profiling of emergency blood samples from matched TBI patients. (**A**,**B**) Chemical classification of identified metabolites in the positive and negative ionization modes. (**C**,**D**) Volcano plots of differential metabolites between the FO and UFO groups. (**E**,**F**) PCA score plots. (**G**) OPLS-DA score plots. (**H**) Hierarchical clustering heatmap of differential metabolites. (**I**) KEGG pathway enrichment analysis. Abbreviations: TBI, traumatic brain injury; FO, favorable outcome; UFO, unfavorable outcome; PCA, principal component analysis; OPLS-DA, orthogonal partial least squares-discriminant analysis; KEGG, Kyoto Encyclopedia of Genes and Genomes.

To investigate the biological mechanisms underlying outcome heterogeneity, blood samples collected in the emergency department from 30 matched pairs (*n* = 60) of male patients with TBI were analyzed using untargeted metabolomics. Differential metabolites in both ionization modes were mainly classified as lipids and lipid-like molecules, organic acids and derivatives, organoheterocyclic compounds, and benzenoids (Figure 3A,B). Volcano plots identified a large number of significantly altered metabolites between favorable and unfavorable outcome groups (Figure 3C,D). PCA showed group separation, while OPLS-DA revealed a more distinct metabolic discrimination between outcome groups (Figure 3E–G). Hierarchical clustering also demonstrated a clearly different metabolite expression pattern (Figure 3H).

KEGG enrichment indicated that differential blood metabolites were mainly involved in lysine degradation, tryptophan metabolism, vitamin digestion and absorption, and protein digestion and absorption (Figure 3I). Representative altered metabolites in the unfavorable outcome group included higher levels of alpha-ketoadipic acid, glutarate, cadaverine, ascorbic acid, vitamin A, pantothenate, citric acid, cholestane-3,7,12,25-tetrol-3-glucuronide, sarcosine, creatinine, 4-acetamidobutanoic acid, platelet-activating factor, and lactate, while L-kynurenine, kynurenic acid, L-2-hydroxyglutaric acid, 3-methoxyanthranilic acid, butyric acid, and piperidine were decreased (Supplementary Figure S1).

### 3.7. Exploratory Untargeted Metabolomics of Intraoperative CSF Samples

**Figure 4:**
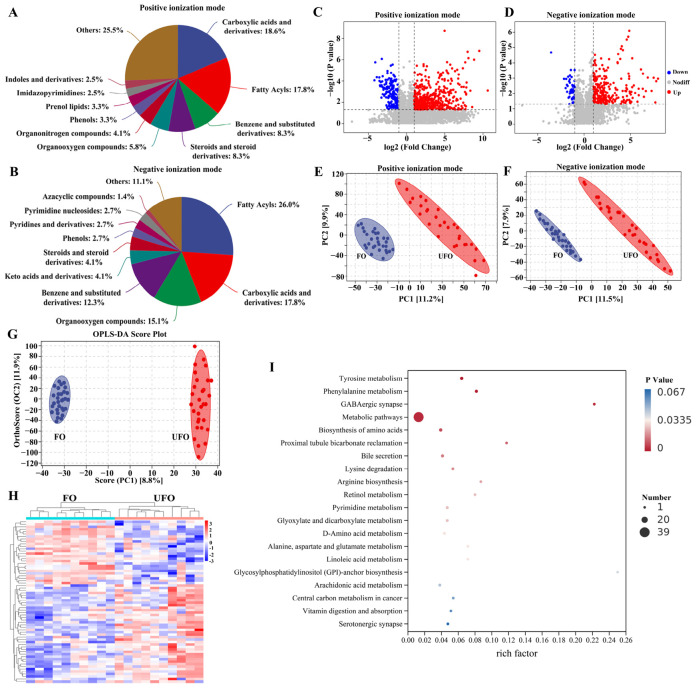
Non-targeted metabolomics profiling of intraoperative cerebrospinal fluid samples from matched TBI patients. (**A**,**B**) Chemical classification of identified metabolites in the positive and negative ionization modes. (**C**,**D**) Volcano plots of differential metabolites between the FO and UFO groups. (**E**,**F**) PCA score plots. (**G**) OPLS-DA score plots. (**H**) Hierarchical clustering heatmap of differential metabolites. (**I**) KEGG pathway enrichment analysis. Abbreviations: TBI, traumatic brain injury; CSF, cerebrospinal fluid; FO, favorable outcome; UFO, unfavorable outcome; PCA, principal component analysis; OPLS-DA, orthogonal partial least squares-discriminant analysis; KEGG, Kyoto Encyclopedia of Genes and Genomes.

In intraoperative CSF from 30 matched pairs (60 cases), untargeted metabolomics similarly demonstrated prominent differences between favorable and unfavorable outcome groups. Differential metabolites were mainly distributed among carboxylic acids and derivatives, fatty acyls, benzene and substituted derivatives, steroids and steroid derivatives, and organic oxygen compounds (Figure 4A,B). Volcano plots and multivariate analyses demonstrated clear intergroup separation in both positive and negative ionization modes (Figure 4C–G), which was further supported by heatmap clustering (Figure 4H).

KEGG enrichment analysis showed that differential CSF metabolites were primarily associated with tyrosine metabolism, phenylalanine metabolism, GABAergic synapse and metabolic pathways (Figure 4I). Relative to the favorable outcome group, the unfavorable outcome group exhibited increased levels of 3,4-dihydroxyphenylpropanoate, ketoleucine, L-arogenate, 18-hydroxycorticosterone, (S)-4-hydroxymandelate, 13(S)-HpOTrE, ergocalciferol, and 11-dehydro-thromboxane B2, while kynurenic acid, vanillylmandelic acid, 3-hydroxyphenylacetic acid, 5-aminopentanoic acid, deoxycytidine, and alpha-tocotrienol were decreased (Supplementary Figure S2).

### 3.8. Integration of Clinical and Metabolomic Findings

The integration of clinical variables with metabolomic signatures revealed complementary information for outcome prediction. Clinical variables provided robust baseline discrimination, while metabolomic profiling identified specific pathway perturbations that may explain the biological basis of these clinical associations.

The strong prognostic performance of lactate (both initial and follow-up) aligns with the metabolomic finding of altered energy metabolism, particularly fatty acid oxidation and glycolysis. The association of GCS-M with outcome, and its persistence in multivariable models, reflects the fundamental importance of neurological examination in capturing injury severity not fully explained by metabolic alterations alone.

The CSF metabolomic findings of neurotransmitter pathway dysregulation (tyrosine metabolism, phenylalanine metabolism, GABAergic synapse) provide mechanistic insights into the neurological dysfunction captured by GCS. Similarly, the prominence of lipid metabolism pathways (linoleic acid, arachidonic acid) in both biofluids suggests that membrane disruption and secondary lipid signaling are central to TBI pathophysiology and may represent therapeutic targets.

## 4. Discussion

### 4.1. Summary of Key Findings and Clinical Implications for Neurocritical Care Management

In this study of 405 TBI patients, we developed prognostic models incorporating readily available admission variables—Rotterdam CT score, lactate, blood glucose, and GCS score—achieving excellent discrimination for 6-month unfavorable outcome (AUC 0.97) and in-hospital mortality (AUC 0.96). Metabolomic profiling of paired blood and CSF revealed coordinated alterations in energy metabolism, lipid signaling, and neurotransmitter function, providing mechanistic context for clinical observations.

These findings carry direct implications for neurocritical care. The model uses universally available admission variables, enabling immediate bedside risk stratification without specialized assays—particularly relevant in resource-limited settings. Integration into neurocritical care protocols could facilitate tiered management: high-risk patients may warrant continuous intracranial pressure monitoring and earlier treatment escalation, while low-risk patients may benefit from conservative management. Serial lactate measurements support real-time treatment algorithms. The metabolomic findings also suggest therapeutic targets; depleted kynurenic acid implicates the tryptophan–kynurenine pathway for pharmacological modulation, while energy metabolism dysregulation supports metabolic resuscitation strategies.

### 4.2. Lactate as a Prognostic Biomarker

Initial lactate predicted both mortality (OR 1.57) and unfavorable outcome (OR 1.81), aligning with growing evidence implicating metabolic derangement in TBI prognosis [3,4]. Lactate elevation reflects tissue hypoxia, mitochondrial dysfunction, and anaerobic glycolysis [13], as well as catecholamine-driven glycogenolysis and impaired clearance. The persistence of lactate elevation in non-survivors suggests ongoing metabolic crisis rather than simply reflecting admission severity. Our optimal initial lactate cut-off of 3.44 mmol/L for mortality prediction (sensitivity 91%, specificity 70%) and 1.75 mmol/L for unfavorable outcome (sensitivity 90%, specificity 71%) provides clinically actionable thresholds, with the higher mortality threshold reflecting more profound metabolic derangement. Our findings are consistent with prior studies demonstrating lactate’s predictive value in TBI [4,14,15,16]. The superior performance in our cohort may reflect our focus on isolated TBI, eliminating confounding from extracranial injuries. Importantly, when evaluated against the IMPACT-Lab proxy model, lactate provided significant incremental prognostic value (NRI = 0.832, IDI = 0.059 for unfavorable outcome; NRI = 1.268, IDI = 0.270 for mortality; all *p* < 0.001). These findings suggest that lactate captures metabolic derangement not fully reflected by established clinical, imaging, and laboratory variables in the IMPACT framework.

### 4.3. Neurological Examination

GCS-M is independently associated with mortality, with each 1-point increment in GCS-M reducing the risk of death by approximately 50%. This underscores that despite advances in biomarkers and imaging, the neurological examination retains irreplaceable prognostic value [17,18]. GCS-M likely captures brainstem functional integrity and corticospinal pathway viability not fully reflected in metabolic derangement alone.

### 4.4. Hyperglycemia and Glycemic Control

Blood glucose independently predicted unfavorable outcome (OR 1.40), consistent with the prior literature. Prisco et al. were among the first to underscore the role of hyperglycemia as a driver of morbidity and mortality in TBI patients admitted to the neurointensive care unit, demonstrating that early hyperglycemia was an independent predictor of in-hospital death [19]. Stress-induced hyperglycemia reflects sympathetic activation and insulin resistance, exacerbating secondary injury through oxidative stress and mitochondrial dysfunction [20,21]. Whether aggressive glycemic control improves outcomes remains controversial [22]; our findings support maintaining euglycemia while avoiding extreme excursions.

### 4.5. Silver Trauma and Age-Specific Considerations

A notable proportion of our cohort (35.1%, *n* = 142) comprised patients aged ≥ 65 years, consistent with the emerging concept of ‘silver trauma’ in TBI. Depreitere et al. highlighted unique considerations in the assessment and management of TBI in older adults, including age-related physiological changes, polypharmacy (particularly antithrombotic use), and distinct injury mechanisms such as falls [23]. In our cohort, older patients (≥65 years) had significantly higher rates of unfavorable outcomes (76.8% vs. 59.7%, *p* = 0.001) and a trend toward higher mortality (17.6% vs. 11.0%, *p* = 0.088). Notably, elderly patients had significantly higher initial lactate levels (median 3.1 vs. 2.6 mmol/L, *p* = 0.013), suggesting more pronounced metabolic derangement at presentation. These findings underscore the need for age-specific prognostic models and management protocols in neurocritical care.

### 4.6. Blood and CSF Metabolomic Signatures

Blood metabolomics revealed enrichment in the tricarboxylic acid cycle (TCA) cycle, glutathione metabolism, hypoxia inducible factor-1 (HIF-1) signaling, and tryptophan metabolism, consistent with acute systemic metabolic stress involving mitochondrial dysfunction and inflammatory reprogramming [24,25]. Elevated lactate and citric acid support disrupted central carbon metabolism, while increased platelet-activating factor mirrors inflammatory activation and microvascular dysfunction [26]. These findings suggest that poor outcome after isolated moderate-to-severe TBI reflects broader systemic metabolic disorganization spanning energy failure, oxidative injury, and neuroinflammation.

CSF metabolomics implicated tyrosine and phenylalanine metabolism, GABAergic synapse, and arachidonic acid metabolism, suggesting disturbances in neurotransmitter synthesis, excitatory–inhibitory balance, and neuroinflammatory lipid mediator production [10]. Increased 11-dehydro-thromboxane B2 and 13(S)-HpOTrE suggest enhanced eicosanoid signaling [27,28], while reduced alpha-tocotrienol indicates depleted antioxidant defenses. CSF sampling heterogeneity (ventricular catheter vs. craniotomy) may introduce variability through differential neuroinflammatory activation, though this reflects real-world clinical practice.

### 4.7. Kynurenine Pathway and Neuroinflammation

Decreased kynurenic acid in both serum and CSF among unfavorable outcome patients suggests impaired neuroprotective tryptophan–kynurenine metabolism. Kynurenic acid is a neuroimmunomodulator upregulated by interferon-gamma and pro-inflammatory cytokines [29,30]. Since it cannot readily cross the blood–brain barrier, its central nervous system presence derives from local astrocytic synthesis [31], positioning the kynurenine pathway as a therapeutic target for neuroprotective strategies.

### 4.8. Metabolomic Signatures and Neurovascular–Axonal Injury

CSF metabolomic alterations may have implications for axonal injury and neurovascular disruption. Dopamine synthesized through the tyrosine hydroxylase pathway modulates neurovascular coupling [32]; aberrant tyrosine metabolite accumulation may reflect disrupted autoregulation [33]. GABAergic perturbation could indicate altered inhibitory neurotransmission and post-traumatic epileptogenesis [25]. Lipid metabolism enrichment is consistent with demyelination and diffuse axonal injury [34]. Future studies incorporating diffusion tensor imaging and perfusion imaging alongside metabolomics would further elucidate these mechanistic connections.

### 4.9. Strengths and Limitations

Several strengths of this study warrant mention. First, the focus on isolated moderate-to-severe TBI minimized confounding from extracranial injuries, allowing more precise characterization of the metabolic alterations specifically attributable to brain injury. Second, the integration of clinical prognostic modeling with untargeted metabolomics in paired blood and CSF samples represents a novel approach that bridges clinical prediction and mechanistic discovery. Third, the use of LASSO regression with bootstrap internal validation provided robust variable selection and overfitting protection.

Limitations include the retrospective single-center design, which may limit generalizability. External validation in independent cohorts is required before clinical implementation. The metabolomic analysis was restricted to males, limiting generalizability to female patients. CSF sampling heterogeneity (ventricular catheter vs. craniotomy) may introduce variability through differential neuroinflammatory activation. Surgical approach was associated with mortality (χ^2^ = 5.41, *p* = 0.020), with craniotomy patients showing higher mortality (19.3% vs. 10.4%), likely reflecting more severe injury burden rather than a causal effect of surgical approach itself.

## 5. Conclusions

Rotterdam CT score, initial lactate, and blood glucose independently predicted 6-month unfavorable outcome (AUC 0.97), while GCS-M, initial lactate, and follow-up lactate predicted in-hospital mortality (AUC 0.96). Metabolomic profiling revealed that unfavorable outcomes are linked to systemic energy–lipid dysregulation and central neuroinflammatory–neurotransmitter disturbances, with the tryptophan–kynurenine axis as a potential mechanistic bridge. These findings support integrating readily available clinical predictors into neurocritical care protocols for early risk stratification and individualized management of TBI patients.

## Figures and Tables

**Table 1:** Demographic characteristics of the cohort (*n* = 405).

	Variable	Number (%)
**Age**	≤30	37 (9.1%)
	31–40	28 (6.9%)
	41–50	52 (12.8%)
	51–60	101 (24.9%)
	61–70	115 (28.4%)
	>71	72 (17.8%)
**Sex**	Male	299 (73.8%)
	Female	106 (26.2%)
**TBI cause**	Road traffic accident	248 (61.2%)
	Ground-level fall	75 (18.5%)
	Fall from height	54 (13.3%)
	Unknown or other	28 (6.9%)
**Admission GCS score**	9–12	179 (44.2%)
	≤8	226 (55.8%)
**Rotterdam CT score**	6	145 (35.8%)
	5	106 (26.2%)
	4	75 (18.5%)
	3	68 (16.8%)
	2	9 (2.2%)
	1	2 (0.5%)
**GOS score**	4–5	139 (34.3%)
	2–3	212 (52.3%)
	1	54 (13.3%)
**Surgery**	Initial Ventricular puncture	270 (66.7%)
	Initial Craniotomy	135 (33.3%)
**Antithrombosis therapy**	Yes	40 (9.9%)
	No	365 (90.1%)
**History of diseases**	No	285 (62.6%)
	Hypertension	89 (19.6%)
	Heart disease	18 (3.9%)
	Cerebral infarction	12 (2.6%)
	Others	11 (2.4%)

TBI: Traumatic Brain Injury, GCS: Glasgow Coma Scale, GOS: Glasgow Outcome Scale.

**Table 2:** Comparative Analysis between Unfavorable and Favorable Outcome Groups (Key variables).

	Unfavorable Outcome	Favorable Outcome	*p*
	N = 266 (65.7%)	N = 139 (34.3%)	
**Age (years, mean ± SD)**	58.5 ± 15.8	53.5 ± 15.1	0.002
**Sex**			0.10
Male	189 (71.1%)	110 (79.1%)	
Female	77 (28.9%)	29 (20.9%)	
**GCS score**	7 [5, 9]	10 [9, 11]	<0.001
**Rotterdam CT score (Sum)**	6 [5, 6]	3 [3, 4]	<0.001
Basal cisterns	2 [2, 2]	0 [0, 1]	<0.001
Midline shift	1 [1, 1]	0 [0, 1]	<0.001
Epidural mass lesion	1 [1, 1]	1 [1, 1]	0.003
Intraventricular blood or tSAH	1 [1, 1]	1 [1, 1]	0.86
**Coagulation parameters**			
International normalized ratio	1.1 ± 0.3	1.0 ± 0.2	0.03
Fibrinogen (g/L)	2.5 ± 1.3	2.8 ± 1.1	0.02
Thrombin time (s)	17.92 ± 4.02	17.2 ± 2.5	0.03
**Hematologic parameters**			
Initial White blood cell (10^9^/L)	16.2 ± 5.7	14.1 ± 5.3	<0.001
Initial Red blood cell (10^12^/L)	4.2 ± 0.6	4.4 ± 0.6	0.004
Initial Hemoglobin (g/L)	129.6 ± 17.9	135.06 ± 16.4	0.003
Initial Hematocrit (%)	38.0 ± 5.3	39.7 ± 4.7	0.002
White blood cell (10^9^/L)	13.5 ± 4.3	12.0 ± 3.8	0.001
Red blood cell (10^12^/L)	3.2 ± 0.7	3.5 ± 0.6	<0.001
Hemoglobin (g/L)	97.6 ± 23.3	107.1 ± 20.9	<0.001
Hematocrit (%)	29.5 ± 6.7	32.0 ± 5.7	<0.001
**Lactate** (mmol/L, mean ± SD)			
Initial Lactate (mmol/L)	4.0 ± 2.2	1.7 ± 1.6	<0.001
Lactate (mmol/L)	3.3 ± 3.4	1.6 ± 1.1	<0.001
**Blood glucose**			
Initial Blood glucose (mmol/L)	9.5 ± 2.8	8.6 ± 2.8	0.003
Blood glucose (mmol/L)	10.0 ± 3.1	8.0 ± 1.7	<0.001

GCS: Glasgow Coma Scale, tSAH: traumatic subarachnoid hemorrhage.

**Table 3:** Multivariable Logistic Regression Analysis for Unfavorable Outcome.

Variables	β (SE)	OR (95% CI)	*p*
Rotterdam CT Score	2.36 (0.27)	10.59 (6.19–18.14)	<0.001
Initial Lactate	0.59 (0.14)	1.81 (1.38–2.36)	<0.001
Blood glucose	0.33 (0.08)	1.40 (1.21–1.64)	<0.001

β: Regression Coefficient, SE: Standard Error, OR: Odds Ratio, CI: Confidence Interval, CT: Computed Tomography.

**Table 4:** ROC Analysis of Predictors for Unfavorable Functional Outcome.

Variables	AUC (95%CI)	Sensitivity	Specificity	Youden Index	Cut Off
Rotterdam CT Score	0.93 (0.91–0.95)	0.86	0.85	0.71	5
Initial Lactate	0.86 (0.81–0.90)	0.90	0.71	0.61	1.75
Blood glucose	0.73 (0.68–0.78)	0.56	0.83	0.39	9.0
Combined Model	0.97 (0.95–0.98)	-	-	-	-

ROC: Receiver Operating Characteristic Curve, AUC: Area Under The Curve, CI: Confidence Interval, CT: Computed Tomography.

**Table 5:** Comparative Analysis between Survivors and Non-survivors (Key variables).

	Survivors	Non-Survivors	*p*
	N = 351 (86. 7%)	N = 54 (13.3%)	
**Age (years, mean ± SD)**	56.2 ± 15.2	60.4 ± 18.3	0.07
**Sex**			0.71
**GCS**	8 [7, 11]	4 [3, 6]	<0.001
**Rotterdam CT score (Sum)**	5 [4, 6]	6 [6, 6]	<0.001
Basal cisterns	2 [1, 2]	2 [2, 2]	<0.001
Midline shift	1 [0, 1]	1 [1, 1]	<0.001
Epidural mass lesion	1 [1, 1]	1 [1, 1]	0.08
Intraventricular blood or tSAH	1 [1, 1]	1 [1, 1]	0.03
**Coagulation parameters**			
International normalized ratio	1.1 ± 0.1	1.2 ± 0.5	0.01
Fibrinogen (g/L)	2.7 ± 1.2	1.8 ± 1.1	<0.001
Thrombin time (s)	17.2 ± 2.3	20.58 ± 7.3	0.001
**Hematologic parameters**			
White blood cell (10^9^/L)	12.7 ± 4.1	14.6 ± 4.4	0.002
Hemoglobin (g/L)	102.6 ± 22.1	89.3 ± 24.8	<0.001
Hematocrit (%)	30.7 ± 6.1	26.3 ± 7.7	<0.001
**Total protein**			
Total protein (g/L)	56.4 ± 6.9	47.6 ± 11.7	<0.001
**Albumin**			
Albumin (g/L)	32.9 ± 5.2	27.7 ± 7.3	<0.001
Delta Albumin	8.4 ± 6.0	13.7 ± 8.5	<0.001
Delta Albumin rate	19.6 ± 12.6	32.1 ± 19.1	<0.001
**Lactate** (mmol/L, mean ± SD)			
Initial Lactate (mmol/L)	2.7 ± 1.9	6.4 ± 2.5	<0.001
Lactate (mmol/L)	2.0 ± 1.5	7.1 ± 5.3	<0.001
**Blood glucose**			
Initial Blood glucose (mmol/L)	9.0 ± 2.6	10.4 ± 3.7	0.01
Blood glucose (mmol/L)	9.0 ± 2.3	11.5 ± 4.7	<0.001

GCS: Glasgow Coma Scale, tSAH: traumatic subarachnoid hemorrhage.

**Table 6:** Multivariable Logistic Regression for in-hospital Mortality.

Variables	β (SE)	OR (95% CI)	*p*
Lactate	0.45 (0.09)	1.57 (1.34–1.88)	<0.001
GCS-M	−0.70 (0.15)	0.50 (0.37–0.66)	<0.001
Initial Lactate	0.45 (0.10)	1.57 (1.31–1.91)	<0.001

β: Regression Coefficient, SE: Standard Error, OR: Odds Ratio, CI: Confidence Interval, GCS-M: Glasgow Coma Scale Motor Score.

**Table 7:** ROC Analysis of Predictors for in-hospital Mortality.

Variables	AUC (95%CI)	Sensitivity	Specificity	Youden Index	Cut Off
Lactate	0.85 (0.78–0.91)	0.69	0.90	0.59	4.15
GCS-M	0.84 (0.79–0.89)	0.78	0.80	0.58	3
Initial Lactate	0.81 (0.75–0.87)	0.91	0.70	0.61	3.44
Combined Model	0.96 (0.94–0.98)	-	-	-	-

ROC: Receiver Operating Characteristic Curve, AUC: Area Under The Curve, CI: Confidence Interval, GCS: Glasgow Coma Scale.

## Data Availability

The raw data supporting the conclusions of this article will be made available by the authors on request.

## Supplementary Materials

The following supporting information can be downloaded at https://www.mdpi.com/article/10.3390/jcm15124592/s1, Figure S1: Relative abundances of representative differentially abundant metabolites in emergency blood samples from matched TBI patients. Figure S2: Relative abundances of representative differentially abundant metabolites in intraoperative cerebrospinal fluid samples from matched TBI patients. Table S1: Comparative Analysis between Unfavorable and Favorable Outcome (All variables). Table S2: IMPACT-Lab proxy model for unfavorable outcome. Table S3. Comparative Analysis between Survivors and Non-survivors (All variables). Table S4. IMPACT-Lab proxy model for in-hospital mortality.

## Abbreviations

The following abbreviations are used in this manuscript:

ALB	Albumin
APTT	Activated Partial Thromboplastin Time
AUC	Area Under the Curve
BBB	Blood–Brain Barrier
CI	Confidence Interval
CNS	Central Nervous System
CRP	C-Reactive Protein
CSF	Cerebrospinal Fluid
CT	Computed Tomography
DCA	Decision Curve Analysis
DDI	D-Dimer
DTI	Diffusion Tensor Imaging
FDP	Fibrin Degradation Products
FIB	Fibrinogen
FO	Favorable Outcome
GABA	Gamma-Aminobutyric Acid
GCS	Glasgow Coma Scale
GCS-E	Glasgow Coma Scale Eye
GCS-M	Glasgow Coma Scale Motor
GCS-V	Glasgow Coma Scale Verbal
GOS	Glasgow Outcome Scale
HB	Hemoglobin
HCT	Hematocrit
HIF-1	Hypoxia-Inducible Factor-1
ICP	Intracranial Pressure
TBI	Traumatic Brain Injury
INR	International Normalized Ratio
IQR	Interquartile Range
KEGG	Kyoto Encyclopedia of Genes and Genomes
LASSO	Least Absolute Shrinkage and Selection Operator
LC-MS	Liquid Chromatography–Mass Spectrometry
MS/MS	Tandem Mass Spectrometry
NPV	Negative Predictive Value
OPLS-DA	Orthogonal Partial Least Squares–Discriminant Analysis
OR	Odds Ratio
PAF	Platelet-Activating Factor
PCA	Principal Component Analysis
PCT	Procalcitonin
PLT	Platelet
PPV	Positive Predictive Value
PT	Prothrombin Time
RBC	Red Blood Cell
ROC	Receiver Operating Characteristic
SD	Standard Deviation
SE	Standard Error
SI	International System of Units
TBI	Traumatic Brain Injury
TCA	Tricarboxylic Acid
TT	Thrombin Time
UFO	Unfavorable Outcome
VIF	Variance Inflation Factor
VIP	Variable Importance in Projection
WBC	White Blood Cell
