# Supplementary material for: Clinical Prognostic Modeling and Paired Blood–CSF Metabolomic Profiling for Outcome Prediction in Isolated Moderate-to-Severe Traumatic Brain Injury: Implications for Neurocritical Care Management
Source: J Clin Med. 2026 Jun 13;15(12):4592. doi: 10.3390/jcm15124592 (PMC13301914; doi:10.3390/jcm15124592)
Supplement: Supplementary file 1 [file jcm-15-04592-s001.zip › jcm-4352219-supplementary.pdf]

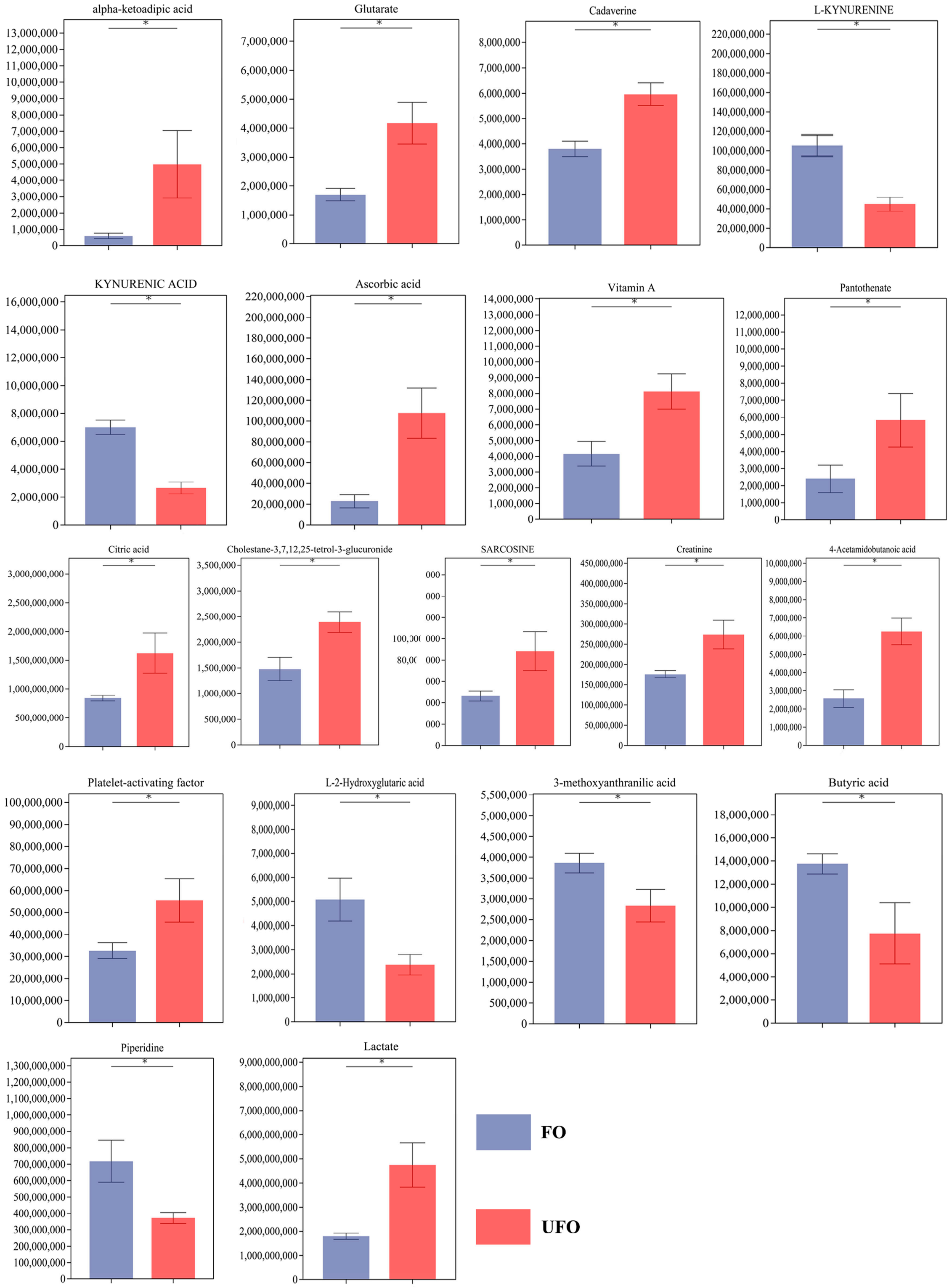

Figure S1. Relative abundances of representative differentially abundant metabolites in emergency blood samples from matched TBI patients.

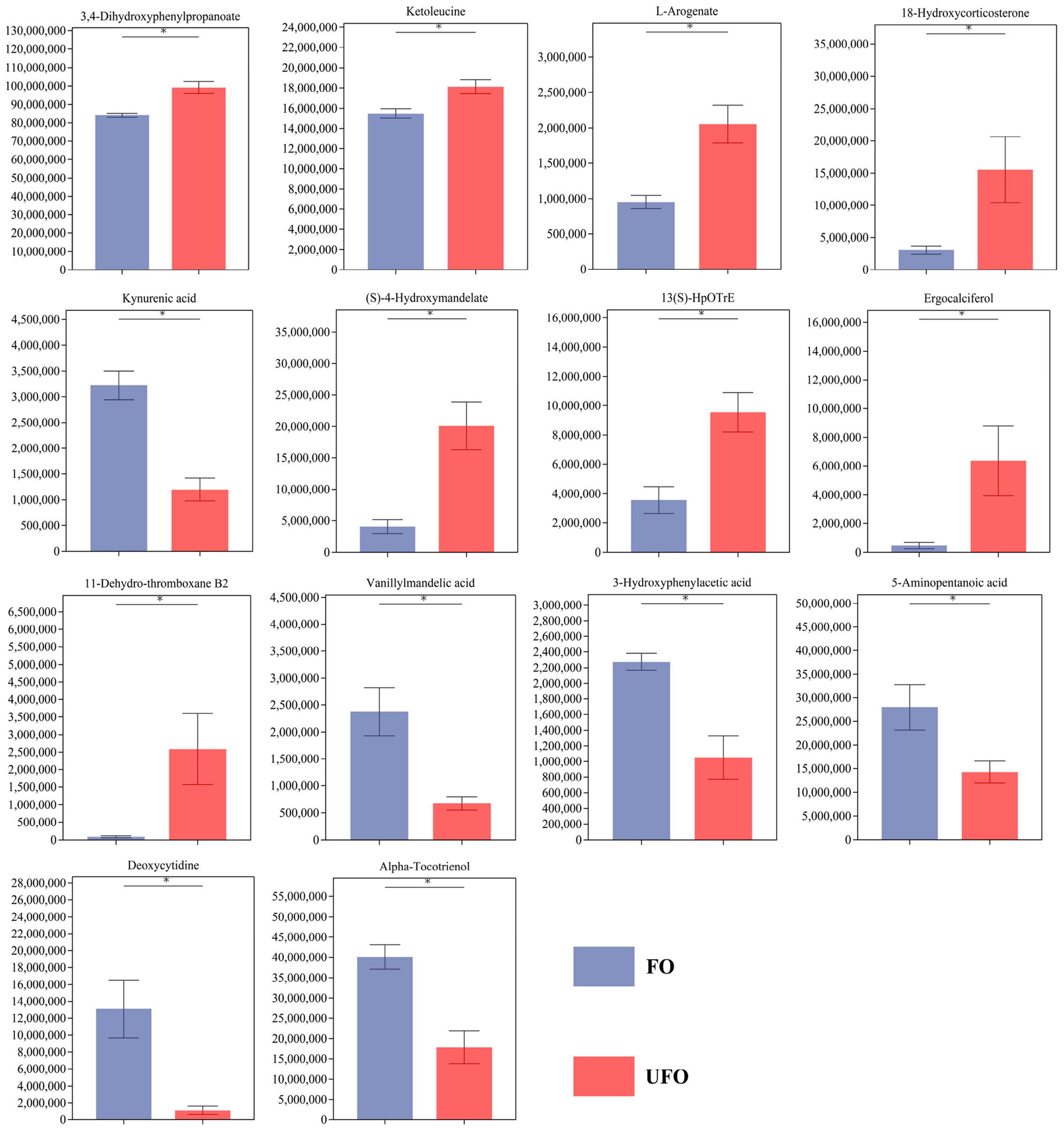

Figure S2. Relative abundances of representative differentially abundant metabolites in intraoper-ative cerebrospinal fluid samples from matched TBI patients.

Table S1. Comparative Analysis between Unfavorable and Favorable Outcome (All variables).

	Unfavorable outcome N = 266 (65.7%)	Favorable outcome N = 139 (34.3%)	*P*
**Age (years, mean ± SD)**	58.5 ±15.8	53.5 ± 15.1	0.002
**Sex**			0.10
Male	189 (71.1%)	110 (79.1%)	
Female	77 (28.9%)	29 (20.9%)	
**TBI cause**			0.12
Road traffic accident	173 (65.0%)	75 (53.9%)	
Ground-level fall	46 (17.3%)	29 (20.9%)	
High-altitude fall	29 (10.9%)	25 (18.0%)	

Unknown or other	18 (6.8%)	10 (7.2%)	
**GCS score**	7 [5, 9]	10 [9, 11]	<0.001
GCS-E	1 [1, 2]	3 [2, 3]	<0.001
GCS-V	1 [1, 2]	3 [1, 3]	<0.001
GCS-M	4 [2, 5]	5 [5, 6]	<0.001
**Rotterdam CT score (Sum)**	6 [5, 6]	3 [3, 4]	<0.001
Basal cisterns	2 [2, 2]	0 [0, 1]	<0.001
Midline shift	1 [1, 1]	0 [0, 1]	<0.001
Epidural mass lesion	1 [1, 1]	1 [1, 1]	0.003
Intraventricular blood or tSAH	1 [1, 1]	1 [1, 1]	0.86
**Surgery**			0.48
Initial Ventricular puncture	181 (68.0%)	89 (64.0%)	
Initial Craniotomy	85 (32.0%)	50 (36.0%)	
**Antithrombosis therapy**			0.43
Yes	29 (10.9%)	11 (7.9%)	
No	237 (89.1%)	128 (92.1%)	
**Coagulation parameters**			
International normalized ratio	1.1 ± 0.3	1.0 ± 0.2	0.03
Prothrombin time (s)	13.1 ± 6.7	12.1 ± 1.5	0.11
Activated partial thromboplastin time (s) (s)	25.7 ± 8.3	24.2 ± 4.3	0.06
Fibrinogen (g/L)	2.5 ± 1.3	2.8 ± 1.1	0.02
Thrombin time (s)	17.92 ± 4.02	17.2 ± 2.5	0.03
D-dimer	37.0 ± 26.5	23.7 ± 23.6	<0.001
Fibrin degradation products (μg/ml)	128.0 ± 163.3	64.6 ± 89.6	<0.001
**Hematologic parameters**			
Initial White blood cell (10^9/L)	16.2 ± 5.7	14.1 ± 5.3	<0.001
Initial Neutrophil counts (10^9/L)	13.5 ± 5.4	12.0 ± 4.7	0.003
Initial Percentage of neutrophils (%)	82.6 ± 12.7	84.4 ± 9.8	0.15
Initial Lymphocyte count (10^9/L)	1.6 ± 1.5	1.4 ± 1.4	0.06
Initial Percentage of lymphocytes (%)	11.0 ± 11.2	9.4 ± 7.9	0.14
Initial Red blood cell (10^12/L)	4.2 ± 0.6	4.4 ± 0.6	0.004
Initial Hemoglobin (g/L)	129.6 ± 17.9	135.06 ± 16.4	0.003
Initial Hematocrit (%)	38.0 ± 5.3	39.7 ± 4.7	0.002
Initial Platelet (10^9/L)	182.8 ± 61.5	181.7 ± 53.0	0.85
White blood cell (10^9/L)	13. 5 ± 4.3	12.0 ± 3.8	0.001
Neutrophil counts (10^9/L)	12.0 ± 6.0	10.5 ± 3.5	0.007
Percentage of neutrophils (%)	86.7 ± 5.5	86.5 ± 5.3	0.77
Lymphocyte count (10^9/L)	1.0 ± 2.0	0.9 ± 0.4	0.31
Percentage of lymphocytes (%)	7.0 ± 3.7	7.4 ± 3.8	0.22
Red blood cell (10^12/L)	3.2 ± 0.7	3.5 ± 0.6	<0.001
Hemoglobin (g/L)	97.6 ± 23.3	107.1 ± 20.9	<0.001
Hematocrit (%)	29. 5 ± 6.7	32.0 ± 5.7	<0.001
Platelet (10^9/L)	128. 7 ± 57.2	139.7 ± 42.9	0.03
Delta Red blood cell	1.0 ± 0.8	0. 9 ± 0.5	0.04
Delta Red blood cell rate	23.7 ± 15.5	20.3 ± 10.8	0.01
Delta Hemoglobin	32.0 ± 24.1	28.1 ± 17.8	0.06
Delta Hemoglobin rate	24.1 ± 16.8	20.6 ± 12.5	0.02
Delta Hematocrit	9.0 ± 6.8	7.6 ± 4.4	0.02
Delta Hematocrit rate	23.0 ± 16.0	19.2 ± 11.1	0.006
**Serum electrolytes**			
Initial Sodium (Na) (mmol/L)	137.7 ± 10.3	138.2 ± 4.3	0.59

Initial Potassium (K) (mmol/L)	3.7 ± 0.5	3.8 ± 0.5	0.07
Initial Calcium (Ca) (mmol/L)	2.2 ± 0.2	2.2 ± 0.2	0.78
Initial Phosphorus (P) (mmol/L)	1.0 ± 0.3	1.00 ± 0.3	0.599
Sodium (Na) (mmol/L)	143.2 ± 5.8	141.4 ± 4.4	<0.001
Potassium (K) (mmol/L)	3.8 ± 0.5	3.9 ± 0.4	0.14
Calcium (Ca) (mmol/L)	2.0 ± 0.2	2.0 ± 0.5	0.07
Phosphorus (P) (mmol/L)	0.9 ± 0.4	0.9 ± 0.4	0.84
**Total protein**			
Initial Total protein (g/L)	69.0 ± 7.4	69.6 ± 7.0	0.43
Total protein (g/L)	54.3 ± 8.9	57.1 ± 6.4	<0.001
Delta Total protein	14.7 ± 10.2	12.5 ± 7.1	0.01
Delta Total protein rate	20.7 ± 13.6	17. 6 ± 9.1	0.006
**Albumin**			
Initial Albumin (g/L)	40.9 ± 5.6	42.0 ± 5.3	0.07
Albumin (g/L)	31.6 ± 5.9	33.3 ± 5.3	0.005
Delta Albumin	9.3 ± 6.8	8.8 ± 6.2	0.42
Delta Albumin rate	21.9 ± 14.8	20.0 ± 13.1	0.19
**Lactate** (mmol/L, mean ± SD)			
Initial Lactate (mmol/L)	4.0 ± 2.2	1.7 ± 1.6	<0.001
Lactate (mmol/L)	3.3 ± 3.4	1.6 ± 1.1	<0.001
**Blood glucose**			
Initial Blood glucose (mmol/L)	9.5 ± 2.8	8.6 ± 2.8	0.003
Blood glucose (mmol/L)	10.0 ± 3.1	8.0 ± 1.7	<0.001
**C-reactive protein** (mg/L)	77.2 ± 46.6	70.3 ± 42.9	0.15
**Procalcitonin** (ng/mL)	1.8 ± 2.0	0.6 ± 0.7	<0.001

TBI: Traumatic Brain Injury, GCS: Glasgow Coma Scale, GCS-M: Glasgow Coma Scale Motor Score, tSAH: traumatic subarachnoid hemorrhage.

Table S2. IMPACT-Lab proxy model for unfavorable outcome.

Model	Variables	AUC	Log-Likelihood
**IMPACT-Lab proxy**	Age + GCS + Rotterdam CT Score + Glucose + Hb	0.957	−102.66
**IMPACT-Lab + Lactate**	Above + Initial Lactate	0.967	−88.78

Incremental Value Metric	Estimate	95% CI / df	*P*-value
**ΔAUC**	+0.010	95% CI: −0.004–0.022	0.17 (ns)
**Likelihood ratio test**	$\chi^2 = 27.76$	df = 1	1.37×10^{-7}
**NRI (overall)**	0.832	95% CI: 0.647–1.00	< 0.001
**NRI (events)**	0.278		
**NRI (non-events)**	0.554		
**IDI**	0.059	95% CI: 0.037–0.080	< 0.001

Lactate coefficient in the IMPACT-Lab + Lactate model

Variable	OR	95% CI	*P*-value
**Age**	1.021	0.995–1.049	0.119
**GCS Motor Score**	1.897	1.211–2.971	0.005
**Rotterdam CT Score**	20.521	9.519–44.238	< 0.001
**Glucose**	1.097	0.942–1.278	0.233
**Hemoglobin**	1.000	0.977–1.024	0.990
**Initial Lactate**	1.826	1.406–2.373	< 0.001

GCS, Glasgow Coma Scale; CT, computed tomography; Hb: Hemoglobin; AUC, area under the curve; CI: Confidence Interval, NRI: Net Reclassification Index; IDI: Integrated Discrimination Improvement; OR: Odds Ratio.

Table S3. Comparative Analysis between Survivors and Non-survivors (All variables).

	Survivors	Non-survivors	*P*
	N = 351 (86.7%)	N = 54 (13.3%)	
**Age (years, mean ± SD)**	56.2 ± 15.2	60.4 ± 18.3	0.07
**Sex**			0.71
Male	258 (73.5%)	41 (75.9%)	
Female	93 (26.5%)	13 (24.1%)	
**TBI cause**			0.26
Road traffic accident	217 (61.8%)	31 (57.4%)	
Ground-level fall	68 (19.4%)	7 (13.0%)	
High-altitude fall	44 (12.5%)	10 (18.5%)	
Unknown or other	22 (6.3%)	6 (11.1%)	
**GCS**	8 [7, 11]	4 [3, 6]	<0.001
GCS-E	2 [1, 3]	1 [1, 2]	<0.001
GCS-V	2 [1, 3]	1 [1, 1]	<0.001
GCS-M	5 [4, 5]	2 [1, 3]	<0.001
**Rotterdam CT score (Sum)**	5 [4, 6]	6 [6, 6]	<0.001
Basal cisterns	2 [1, 2]	2 [2, 2]	<0.001
Midline shift	1 [0, 1]	1 [1, 1]	<0.001
Epidural mass lesion	1 [1, 1]	1 [1, 1]	0.08
Intraventricular blood or tSAH	1 [1, 1]	1 [1, 1]	0.03
**Surgery**			0.01
Initial Ventricular puncture	242 (69.0%)	28 (51.9%)	
Initial Craniotomy	109 (31.0%)	26 (48.1%)	
**Antithrombosis therapy**			0.41
Yes	33 (9.4%)	7 (13.0%)	
No	318 (90.6%)	47 (87.0%)	
**Coagulation parameters**			
International normalized ratio	1.1 ± 0.1	1.2 ± 0.5	0.01
Prothrombin time (s)	12.6 ± 5.6	14.0 ± 4.5	0.07
Activated partial thromboplastin time (s)	24.6 ± 4.1	29.2 ± 16.3	0.04
Fibrinogen (g/L)	2.7 ± 1.2	1.8 ± 1.1	<0.001
Thrombin time (s)	17.2 ± 2.3	20.58 ± 7.3	0.001
D-dimer	29.5 ± 25.4	51.5 ± 24.3	<0.001
Fibrin degradation products (μg/ml)	93.7 ± 131.8	187.7 ± 196.3	0.001
**Hematologic parameters**			
Initial White blood cell (10^9/L)	15.3 ± 5.6	16.5 ± 5.6	0.17
Initial Neutrophil counts (10^9/L)	13.0 ± 5.2	13.2 ± 5.5	0.84
Initial Percentage of neutrophils (%)	83.9 ± 11.1	78.8 ± 14.6	0.02
Initial Lymphocyte count (10^9/L)	1.4 ± 1.4	2.2 ± 2.0	0.01
Initial Percentage of lymphocytes (%)	9.8 ± 9.2	15.0 ± 14.7	0.02
Initial Red blood cell (10^12/L)	4.3 ± 0.6	4.3 ± 0. 6	0.54
Initial Hemoglobin (g/L)	131.2 ± 17.7	133.35 ± 16.9	0.39

Initial Hematocrit (%)	38.5 ± 5.2	39.3 ± 5.0	0.29
Initial Platelet (10^9/L)	181.7 ± 59.6	187.20 ± 52.1	0.52
White blood cell (10^9/L)	12.7 ± 4.1	14.6 ± 4.4	0.002
Neutrophil counts (10^9/L)	11.3 ± 5.5	12.4 ± 3. 7	0.16
Percentage of neutrophils (%)	86.7 ± 5.3	86.0 ± 6.4	0.37
Lymphocyte count (10^9/L)	0.9 ± 1.7	1.2 ± 1.2	0.35
Percentage of lymphocytes (%)	7.1 ± 3.4	7.5 ± 5.6	0.61
Red blood cell (10^12/L)	3.4 ± 0.7	2.9 ± 0.9	<0.001
Hemoglobin (g/L)	102.6 ± 22.1	89.3 ± 24.8	<0.001
Hematocrit (%)	30.7 ± 6.1	26.3 ± 7.7	<0.001
Platelet (10^9/L)	137.1 ± 50.0	102.4 ± 61.6	<0.001
Delta Red blood cell	0.9 ± 0.6	1.4 ± 0.9	<0.001
Delta Red blood cell rate	21.1 ± 12.7	31.7 ± 19.2	<0.001
Delta Hemoglobin	28.5 ± 20.2	44.1 ± 28.6	<0.001
Delta Hemoglobin rate	21. 5 ± 14.4	32.2 ± 19.0	<0.001
Delta Hematocrit	7.81 ± 5.3	13.0 ± 8.8	<0.001
Delta Hematocrit rate	20.1 ± 13.0	32.1 ± 19.8	<0.001
**Serum electrolytes**			
Initial Sodium (Na) (mmol/L)	137.9 ± 9.2	138.3 ± 4.1	0.73
Initial Potassium (K) (mmol/L)	3.7 ± 0.5	3.6 ± 0.5	0.04
Initial Calcium (Ca) (mmol/L)	2.2 ± 0.2	2.1 ± 0.2	0.15
Initial Phosphorus (P) (mmol/L)	1.0 ± 0.3	1.0 ± 0.3	0.12
Sodium (Na) (mmol/L)	142.0 ± 4.9	146.1 ± 7.3	<0.001
Potassium (K) (mmol/L)	3.8 ± 0.43	3.9 ± 0.6	0.42
Calcium (Ca) (mmol/L)	2.0 ± 0.37	1. 9 ± 0.3	0.04
Phosphorus (P) (mmol/L)	0.9 ± 0.3	1.0 ± 0.7	0.10
**Total protein**			
Initial Total protein (g/L)	69.1 ± 7.1	69.7 ± 8.2	0.55
Total protein (g/L)	56.4 ± 6.9	47.6 ± 11.7	<0.001
Delta Total protein	12.7 ± 7. 9	22.1 ± 12.9	<0.001
Delta Total protein rate	17.9 ± 10.2	31.0 ± 17. 7	<0.001
**Albumin**			
Initial Albumin (g/L)	41.3 ± 5.5	41. 6 ± 5.5	0.82
Albumin (g/L)	32.9 ± 5.2	27.7 ± 7.3	<0.001
Delta Albumin	8.4 ± 6.0	13.7 ± 8.5	<0.001
Delta Albumin rate	19.6 ± 12.6	32.1 ± 19.1	<0.001
**Lactate** (mmol/L, mean ± SD)			
Initial Lactate (mmol/L)	2.7 ± 1.9	6.4 ± 2.5	<0.001
Lactate (mmol/L)	2.0 ± 1.5	7.1 ± 5.3	<0.001
**Blood glucose**			
Initial Blood glucose (mmol/L)	9.0 ± 2.6	10.4 ± 3.7	0.01
Blood glucose (mmol/L)	9.0 ± 2.3	11.5 ± 4.7	<0.001
**C-reactive protein** (mg/L)	74.6 ± 43.6	76.5 ± 56.0	0.81
**Procalcitonin** (ng/mL)	1.2 ± 1.6	2.6 ± 2.0	<0.001

TBI: Traumatic Brain Injury, GCS: Glasgow Coma Scale, GCS-M: Glasgow Coma Scale Motor Score, tSAH: traumatic subarachnoid hemorrhage.

Table S4. IMPACT-Lab proxy model for in-hospital mortality.

Model	Variables	AUC	Log-Likelihood
**IMPACT-Lab proxy**	Age + GCS + Rotterdam CT Score + Glucose + Hb	0.875	−128.55

**IMPACT-Lab + Initial Lactate**	Above + Initial Lactate	0.935	−104.44
**IMPACT-Lab + Both Lactate**	Above + Follow-up Lactate	0.960	−86.11

Comparison	χ^2	df	*P*-value
**IMPACT-Lab vs + Initial Lactate**	48.22	1	3.80×10^{-12}
**IMPACT-Lab vs + Both Lactate**	84.87	2	3.72×10^{-19}

Incremental Value Metric	IMPACT-Lab + Both Lactate vs IMPACT-Lab alone
**ΔAUC**	+0.084
**NRI (overall)**	1.268
**IDI**	0.270

Lactate coefficients in the IMPACT-Lab + Both Lactate mortality model:

Variable	OR	95% CI	*P*-value
**Age**	1.051	1.016–1.087	0.004
**GCS Motor Score**	0.374	0.234–0.597	< 0.001
**Rotterdam CT Score**	0.932	0.418–2.075	0.863
**Glucose**	1.016	0.863–1.196	0.846
**Hemoglobin**	1.022	0.993–1.051	0.141
**Initial Lactate**	1.578	1.288–1.932	< 0.001
**Follow-up Lactate**	1.608	1.342–1.927	< 0.001

GCS, Glasgow Coma Scale; CT, computed tomography; Hb: Hemoglobin; AUC, area under the curve; CI: Confidence Interval, NRI: Net Reclassification Index; IDI: Integrated Discrimination Improvement; OR: Odds Ratio.
